# Effects of Leucine Administration in Sarcopenia: A Randomized and Placebo-controlled Clinical Trial

**DOI:** 10.3390/nu12040932

**Published:** 2020-03-27

**Authors:** Francisco M. Martínez-Arnau, Rosa Fonfría-Vivas, Cristina Buigues, Yolanda Castillo, Pilar Molina, Aldert J. Hoogland, Femke van Doesburg, Leo Pruimboom, Julio Fernández-Garrido, Omar Cauli

**Affiliations:** 1 Department of Physiotherapy, University of Valencia, 46010 Valencia, Spain; francisco.m.martinez@uv.es; 2 Frailty and Cognitive Impairment Research Group (FROG), University of Valencia, 46010 Valencia, Spain; rosa.fonfria@uv.es (R.F.-V.); cristina.buigues@uv.es (C.B.); julio.fernandez@uv.es (J.F.-G.); 3 Department of Nursing, University of Valencia, 46010 Valencia, Spain; 4 La Saleta, Armonea Group, 46015 Valencia, Spain; supbetera@lasaleta.com (Y.C.); pmolina@lasaleta.com (P.M.); 5 Natura Foundation, 3281 NC Numansdorp, The Netherlands; a.hoogland@bonusan.nl (A.J.H.); F.vanDoesburg@naturafoundation.com (F.v.D.); 6 University of Groningen, University Medical Center Groningen (UMCG), 9712 CP Groningen, The Netherlands; cpni.pruimboom@icloud.com; 7 Psychoneuroimmunologie (PNI) Europe, 2491 The Hague, The Netherlands

**Keywords:** respiratory muscles, sarcopenia, muscle mass, muscle strength, elderly, nutrition

## Abstract

Treating sarcopenia in older individuals remains a challenge, and nutritional interventions present promising approaches in individuals that perform limited physical exercise. We assessed the efficacy of leucine administration to evaluate whether the regular intake of this essential amino acid can improve muscle mass, muscle strength and functional performance and respiratory muscle function in institutionalized older individuals. The study was a placebo-controlled, randomized, double-blind design in fifty participants aged 65 and over (ClinicalTrials.gov identifier NCT03831399). The participants were randomized to a parallel group intervention of 13 weeks’ duration with a daily intake of leucine (6 g/day) or placebo (lactose, 6 g/day). The primary outcome was to study the effect on sarcopenia and respiratory muscle function. The secondary outcomes were changes in the geriatric evaluation scales, such as cognitive function, functional impairment and nutritional assessments. We also evaluated whether leucine administration alters blood analytical parameters and inflammatory markers. Administration of leucine was well-tolerated and significantly improves some criteria of sarcopenia in elderly individuals such as functional performance measured by walking time (*p* = 0.011), and improved lean mass index. For respiratory muscle function, the leucine-treated group improved significantly (*p* = 0.026) in maximum static expiratory force compared to the placebo. No significant effects on functional impairment, cognitive function or nutritional assessment, inflammatory cytokines IL-6, TNF-alpha were observed after leucine administration compared to the placebo. The use of l-leucine supplementation can have some beneficial effects on sarcopenia and could be considered for the treatment of sarcopenia in older individuals.

## 1. Introduction

Sarcopenia refers to the loss of skeletal muscle mass and strength associated to aging and it can be also found in several disorders. Sarcopenia occurring with aging has a gradual course and is widely associated to an increased risk of adverse outcomes such as physical disability, poor quality of life and mortality [1,2]. Its prevalence has been estimated at 5%–13% in people aged 60–70 and as high as 50% in those aged over 80 [3]. Several reports performed in older populations showed that individuals have an increased risk of falls [4,5], frailty, loss of ability to performed basic activities [6], and type-II diabetes [7,8], all of which all impair the quality of life and determine high rates of morbidity and premature mortality. 

A recent study conducted in the US population, based on the National Health and Nutrition Examination Survey (NHANES) survey, demonstrated that muscle mass is a significant predictor of longevity for all-cause mortality [6] taking into account men and women over 55 or 65 years of age and over, respectively. One of the main pathway by which the loss of muscle mass contributes to the appearance or the progression of other diseases is the alteration of protein turnover and metabolism in the skeletal muscle tissue [9,10]. In addition, other factors such as the reduced anabolic response to protein feeding during aging [11,12], contributes to the loss of muscle mass in individuals with sarcopenia. Confirming this, metabolic studies showed that older individuals aged 65 years and over require approximately 2% more protein intake per meal to maximize muscle protein synthesis [13]. Furthermore, globally, only 40% of older adults meet the recommended daily protein intake and 10% of older women do not even meet the estimated minimal average requirement [14,15].

One of the nutritional strategies that has been shown to be potentially useful in increasing protein synthesis at the muscle level is supplementation with leucine, an essential branched chain amino acid that has important regulatory actions in the muscles that are mediated, at least partially, by the mammalian target of the rapamycin pathway [9,10]. Leucine modulates the rate of protein turnover in skeletal muscles by decreasing proteolysis and increasing protein synthesis. Several studies have shown that administration of leucine can improve protein synthesis in muscles [16,17,18,19,20]. A recent clinical trial demonstrated that co-administration of 1.5 g of free leucine via the oral route with a single 15-g bolus of protein further increased the rate of muscle protein during recovery from resistance-based physical exercise in older men, suggesting leucine-effects are synergic with those achieved by physical exercise at least under some experimental conditions [12]. In addition, leucine may also stimulate the release of insulin by the pancreatic beta cells [21], and thus its beneficial effect may also improve glucose uptake in skeletal muscle, increasing the anabolic signal in skeletal muscle and contributing positively to the maintenance of muscle mass.

In view of the above, administration of leucine-containing supplements has been proposed as promising approach for treating sarcopenia at least in community-dwelling older individuals. Most randomized placebo controlled trials (RCTs) have included interventions using whey protein as a supplement, because these contain large amounts of leucine (approximately 13 g leucine/100 g protein [13]). However, scientific evidence regarding the effect of leucine alone in sarcopenia is very scarce [11,14,15,16]. We performed an RCT in order to ascertain whether the administration of leucine alone is effective in the treatment of sarcopenia and respiratory muscle function in older institutionalized individuals, a subgroup of older individuals with huge amount of comorbidity who are seldom evaluated in RCTs using leucine supplementation.

The main objectives of the RCT were: (1) to analyze the effects of administration of oral leucine on sarcopenia criteria, e.g., muscle mass, strength, and functional activity in older individuals; (2) to evaluate its effects on respiratory muscle function using, as indicators, the maximum static respiratory pressures at the mouth, inspiratory (MIP) and expiratory (MEP), and the peak expiratory flow (PEF); (3) to investigate the effects of leucine supplementation on other secondary outcomes included in integrative psychogeriatric evaluation such as cognitive function, the ability to perform the basic activities of life, and nutritional assessment.

## 2. Materials and methods

### 2.1. Study Population

The participants of the RCT were men and women living in nursing homes (Valencia region, Spain). All individuals fulfilled the inclusion criterion to be able to walk 6 m and were 65 years or over. The exclusion criteria were severe cognitive impairment precluding an understanding of the questions included in the questionnaires and scales, poor controlled psychiatric disease such as schizophrenia or blindness, acute infections or known cancer. The sample size was estimated as a function of the differences in the measurements between the groups that should be detected (=1 sarcopenia criterion among the six parameters of sarcopenia muscle strength, muscle mass, walking time, maximum static inspiratory (MIP) or expiratory (MEP) pressures at the mouth, and peak expiratory flow (PEF)), the standard deviation of those values, the p-value (0.05) and the power (90%). In such old institutionalized individuals like those enrolled in the RCT (mean age 79 years old), our experience showed that there is a variability range 10%–30% of dropouts in experimental studies performed in nursing homes. For this reason, we assumed a power of 90% in order to minimize the impact of an eventual high rate of dropouts during the study.

As a result, to achieve 90% power with a two-sided 5% level of significance, and to detect a minimum difference of one sarcopenia parameter (we measured six sarcopenia parameters) between the placebo and leucine groups, we calculated that a sample size of a total of 44 patients would enter this two-treatment crossover study [22]. We therefore planned to recruit 50 volunteers to allow for dropouts of people that could not complete the investigation. The study was approved by the Ethics Committee of University of Valencia (Valencia, Spain) (H1524420647893 date 5 July 2018). Written informed consent was obtained from all participants. The clinical trial was registered on ClinicalTrials.gov with the identifier number NCT03831399.

### 2.2. Intervention

The tested compounds, L-leucine and the placebo (lactose), in powder form, were always stored in bottles and kept at room temperature. Nurses or the participants themselves under nurse supervision dissolved two spoons of L-leucine (6 g/day) or placebo (lactose, 6 g/day) in a glass of water or juice, and it was administered in the morning shortly after breakfast (9–10 a.m.) (approximately 3 g) and in the afternoon at 4–5 p.m. (approximately 3 g). The participants were instructed to report any secondary effects of gastrointestinal alteration and to maintain their usual dietary habits. The participants were interviewed on a weekly basis about any eventual adverse effects, including the most common gastro-intestinal side effects such as dyspepsia, flatulence, and changes in stool characteristics (consistency and frequency). Compliance with leucine or placebo administration was assessed on the basis of a diary completed daily by nurses working in the nursing homes. The nurses recorded whether people did not wish to drink the supplement or if there were any problems at the time of administration, for example, if individuals fell asleep at the time of administration or were outside the nursing home for any reason at the time of administration. Adequate compliance was defined as having consumed 10 of the possible 14 administrations per week. Percent compliance was calculated by determining the number of dosage units consumed, divided by the number expected to have been taken and multiplied by 100. The diets in the nursing homes are designed and constantly supervised by a team of nutritionists/dieticians.

### 2.3. Measurement of Sarcopenia

Sarcopenia was measured according to the guidelines of the European Working Group on Sarcopenia in Older People (EWGSOP) [1], and can be assessed by means of indirect measures of muscle function and muscle mass, such as low walking speed (≤0.8 m/s walking 4.6 m), handgrip strength assessed by dynamometry (men ≤ 30 kg/m^2^ and women ≤ 20 kg/m^2^) and the loss of lean mass calculated using the equation formulated by Janssen [17] based on the resistance values obtained in the whole body bioimpedance assessment (women ≤ 5.5 kg/m^2^ and men ≤ 7.25 kg/m^2^). These lean mass values can be adjusted to different populations, with cut-off values for the Spanish population off 8.31 kg/m2 for men and 6.68 kg/m2 for women [10]. We assessed body composition by bioelectrical impedance analysis (BIA) with a Tanita BF-300 device. The BIA measure was performed with a standard technique using a single frequency of 50 KHz and the placement of four electrodes in a distal position (four electrodes at feet). The participant was measured while in a standing position. The values of reactance and resistance were then recorded once the patient was stabilized. Muscle mass was calculated using the formula of Janssen et al. [17]: muscle mass (kg) = [(height^2^ / R × 0.401) + (3.825 × sex) + (−0.701 × age) + 5102, where height is expressed in cm, R in ohms, age in years and female sex has a value of zero and males a value of one. The muscle mass index (MMI) is defined as the muscle mass a person has, corrected by body surface area (muscle mass/height^2^). Muscular strength was measured in the dominant hand with a handgrip dynamometer (Saehan Smedley Hand Dynamometer^®^), the test was always repeated three times during a 5 min period, with the mean value of the trials being recorded.

For the assessment of muscle respiratory function, a spirometric assessment was chosen to collect the main pulmonary volumes and flows, as well as an assessment of the maximum respiratory pressures measured in the mouth produced by the contraction of the respiratory muscles to gather respiratory muscle strength.

In order to perform spirometry, the patient was placed in a sitting position, with their back resting on a backrest and with nasal forceps to avoid air leaks. The maneuver was explained in detail, and the patient was instructed to start with maximum inspiration until reaching total lung capacity, followed by maximum forced exhalation until no more air could be expelled. Three repetitions of the maneuver were performed, with a pause of 1 min between them, and the highest value of the three repetitions was recorded. The spirometric assessment followed the standardized recommendations of the European Respiratory Society [18].

The data obtained were volumes and forced pulmonary capacities: forced vital capacity (FVC), forced expiratory volume in the first second (FEV1), FEV1/FVC, forced expiratory volume in diameter tracks smaller than 1 mm (FEV2575, FEV25, FEV50, FEV75) and peak expiratory flow (PEF). Absolute values and relative values were obtained with respect to a sample of similar healthy subjects.

Maximum static respiratory pressures at the mouth, inspiratory (MIP) and expiratory (MEP), were assessed for the measurement of respiratory muscle strength. Measurements of maximum static inspiratory (MIP) or expiratory (MEP) pressures at the mouth enable a simple assessment of global respiratory muscle strength in a clinical setting. The tests are volitional and require the subject’s full cooperation to achieve maximum isometric effort. MIP was measured at residual volume and MEP at total lung capacity to record the maximum value of three maneuvers that vary by less than 10%. The standardized regulations for this test were followed [19,20]. To achieve the best possible performance, patients were thoroughly instructed by a researcher and then proceeded to begin the test. At least three trials were performed (with less than 10% variation between trials or they were discarded) and the highest result was recorded.

### 2.4. Geriatric Assessment

We performed a complete psycho-geriatric assessment at baseline and after completion of the RCT. Barthel index was measured as a surrogate of the ability to perform the basic activities of daily living, and the score range is a 0–100 point index, where zero is total dependence and 100 is total independence [21]. The Mini Mental State Examination (MMSE) test was used to estimate cognitive function [23] with a score range of 0–30, i.e., with the highest scores indicating better cognitive performance. Nutritional status was evaluated by the Mini Nutritional Assessment (MNA) [24], a tool commonly used in the geriatric population which has a score range of 0–30, with a score < 24 indicating malnutrition risk. The general health status was estimated using the Charlson Comorbidity Index [25] adjusted for age. We determined the extent of physical activity by the self-administered International Physical Activity Questionnaire (IPAQ) whose questions ask about the time spent being physically active in the last 7 days.

### 2.5. Blood Analytical Parameters

Blood samples were taken in fasting conditions between 7:30 a.m and 9 a.m. Blood samples were collected through two vacutainer tubes (one for plasma and the other for serum) containing EDTA. After extraction, the samples were centrifuged at 1500 rpm for 10 min at room temperature. The supernatants were subsequently aliquoted and stored at −20 °C until analysis. Residential center-controlled blood extractions were used for all other analytical determinations. The haemogram and several biochemical parameters were measured (glucose, urea, urate, cholesterol, triglycerides, total proteins, creatinine, calcium, sodium, potassium, transaminases, vitamin D (as 25OHD), C-reactive protein) in clinical laboratories belonging to local public health centers. The concentration of cytokines TNF-α and IL-6 in plasma was performed by using a commercial enzyme-linked immunosorbent assay kit according to the manufacturer’s instructions (TNF-α (ab100654), IL-6 (ab100572), Human ELISA Kit, Abcam^®^). All the cytokine measurements were conducted in duplicate and on the same day to minimize assay variance.

### 2.6. Statistical Analysis

Descriptive statistics include mean and standard deviation for quantitative variables and frequency for qualitative variables. The normal data distribution of each variable was estimated with the Shapiro–Wilk test. The non-parametric Mann–Whitney U test was performed to verify any differences between the two experimental groups for quantitative variables. In order to analyze how the changes in walking time were related to the amount of physical activity, we used the non-parametric correlation Spearman test (the strength identified by the Spearman’s *rho* coefficient and p value). In order to estimate the magnitude of the significant changes (*p* < 0.05) between the two experimental group we calculated Cohen’s d and effect size r (small (d = 0.2), medium (d = 0.5), and large (d ≥ 0.8)). Statistical significance was set at *p* < 0.05, and statistical analysis was performed with the SPSS 24.0 (SPSS Inc., Chicago, IL, USA) software package.

## 3. Results

### 3.1. Design and Study Population

The mean age of the participants was 78.9 ± 7.9 (SD) (range 65–93 years). No significant difference (*p* = 0.81) was observed between the mean age of individuals in the leucine group (78.4 ± 8.4) compared to the placebo group (79.0 ± 7.6). No significant difference (*p* = 0.97) was found regarding the distribution of sexes in the experimental groups. The mean energy intake at baseline from the dieticians’ reports was 1770 ± 196 kcal/day for the leucine group and 1758 ± 168 kcal/day for the placebo group, with no differences between groups (*p* = 0.96). At 6 weeks of treatment, mean energy intake was 1632 ± 188 kcal/day for the leucine group and 1690 ± 204 kcal/day for the placebo group with no differences between groups (*p* = 0.83). After completion of the trial mean energy intake was 1694 ± 152 kcal/day for the leucine group and 1707 ± 195 kcal/day for the placebo group with no differences between groups (*p* = 0.91).

The primary outcome measured was the effect of treatment on sarcopenia criteria, e.g., lean mass, muscle strength and physical performance and respiratory muscle function, e.g., expressed by the maximum static respiratory pressures at the mouth during inspiration (MIP) and expiration (MEP). The secondary outcome was the effect on functional activity assessed by the Barthel Index, cognitive impairment by the Mini Mental examination score and nutritional status by Mini.

Nutritional Assessment (MNA). We also analyzed if the treatment influenced the values of common blood analytical parameters and the hemogram. The participants were enrolled and all clinical (sarcopenia and geriatric assessments) and analytical (blood analyses) evaluations were performed at baseline (week zero). Randomization was carried out after the enrolment and baseline evaluations were completed. The intervention took place between June and September 2019. The participants were selected based on inclusion and exclusion criteria (see Study Population). The baseline evaluation was performed approximately 5 weeks before randomization due to ethical protocol issues in the nursing homes. During the initial week, researchers met with staff from the three participating nursing homes to explain the study aims and experimental protocol. In the second week, researchers met with residents of the nursing homes who fulfilled the inclusion criteria. At week three, researchers met with the relatives of residents of the nursing homes that fulfilled the inclusion criteria. Informing families of residents from the nursing homes was a necessary requirement prior to commencing any new intervention. Within the last two weeks prior to the commencement of the study, the compounds were prepared, bottles containing the test compounds were masked labelled, and then these were shipped to the nursing homes (from the Netherlands to Spain). The treatment with leucine or placebo lasted 13 weeks. All participants were re-evaluated (sarcopenia, geriatric assessments and blood sample analyses) within 1 month of completion of the study.

### 3.2. Dropouts, Safety and Compliance

**Figure 1:**
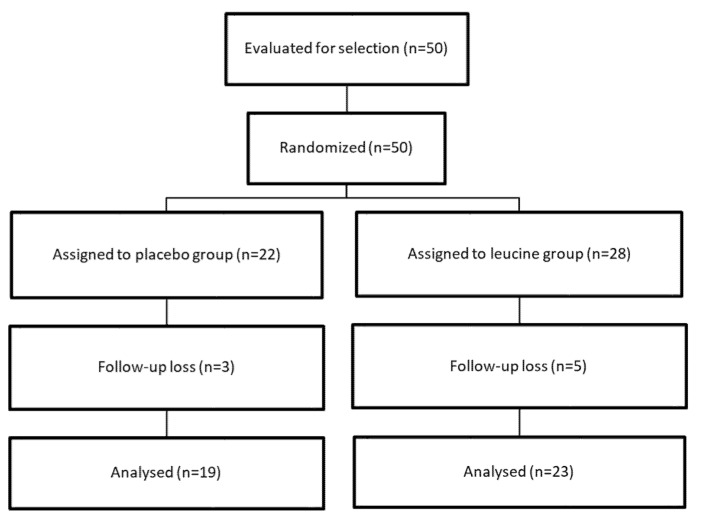
Study flowchart.

The flowchart of the RCT is shown in Figure 1. A total of forty-two participants completed the study (86.0%) (14 men and 29 women). Dropout rates were not significantly (*p* = 0.71) different between groups (five participants in the leucine group and three participants in the placebo group stopped the treatment and did not wish to participate for unknown reasons). No significant changes (*p* = 0.84) in drug administration prescribed for the comorbidities were observed between the two groups during the trial. In those individuals completing all the study protocol and included in the analysis, intervention compliance was high (91.5% in the leucine group and 93.5% in the placebo group) from baseline to follow-up, and did not differ significantly between groups.

No adverse events were recorded among the participants who dropped out of the study. The two experimental groups (leucine and placebo) were not significantly different at baseline (see Table 1) except for the lean mass index, which was significantly lower in the leucine group compared to the control group (*p* = 0.02).

### 3.3. Effect of Leucine Administration on Sarcopenia Criteria

**Figure 2:**
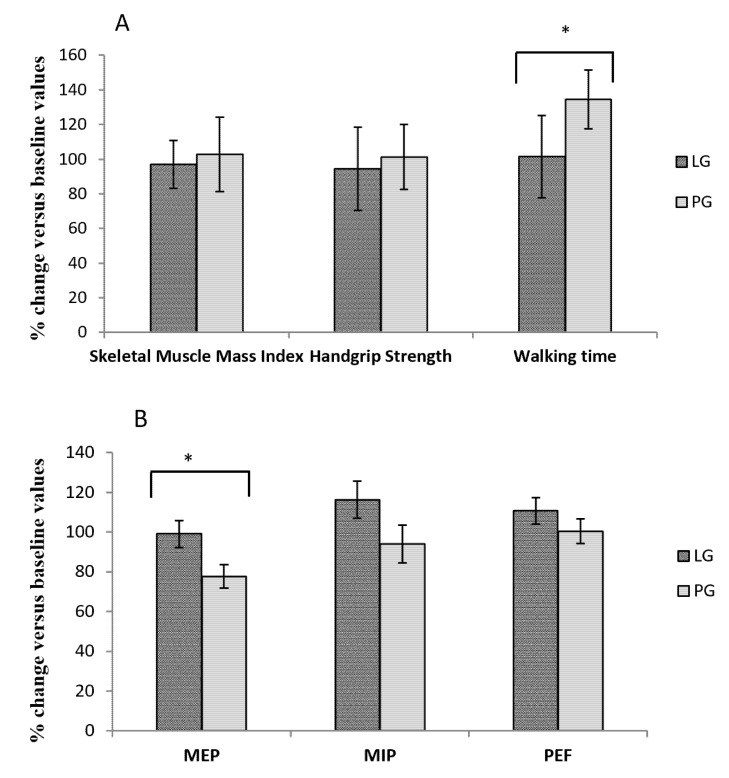
Effect of Leucine administration on sarcopenia criteria (**A**) and muscle respiratory sarcopenia (**B**). (**A**) Comparison of percentage changes compared to baseline values after leucine or placebo treatment for muscle mass index, handgrip strength and walking time * *p* = 0.011. (**B**) Comparison of percentage changes compared to baseline values after leucine or placebo treatment for respiratory muscle function * *p* = 0.026. Leucine group (LG); placebo group (PG); maximum static inspiratory (MIP) and expiratory (MEP) respiratory pressures at the mouth; peak expiratory flow (PEF).

Handgrip strength and walking time values did not differ between the groups at baseline (leucine group: 43.03 ± 4.10 vs. placebo group: 35.97 ± 0.41; *p* = 0.242 and leucine group 10.7 ± 2.85 vs. placebo group: 10.43 ± 2.42; *p* = 0.944 respectively). The only differences were in in the muscle mass index, with greater muscle mass in the control group compared to the leucine group (leucine group: 8.3 ± 1.8 vs. placebo group: 10.4 ± 3.4; *p* = 0.02, Cohen’s d = 0.77, effect size r = 0.36). Following the intervention, there was no difference between groups for the muscle mass index: leucine group: 8.3 ± 1.7 and placebo group: 9.9 ± 3.4; *p* = 0.08, and in the handgrip strength values: leucine group: 16.4 ± 10.7 vs. placebo group: 18.3 ± 8.8; *p* = 0.55 and walking time values were: leucine group: 8.27 ± 1.10 vs. placebo group: 9.92 ± 1.05; *p* = 0.285. The changes induced by leucine administration in sarcopenia parameters, e.g., skeletal muscle mass index, handgrip strength and walking time are presented in Figure 2A.

There was a significant difference in walking time compared to baseline levels, expressed as 100% between the leucine and placebo groups, with an increase in the time required to complete the distance in the placebo group (leucine group: 101.43 ± 6.00 vs. placebo group: 134.41 ± 10.47; *p* = 0.011, Cohen’s d = 3.88, effect size r = 0.89). There were no significant differences between the groups in skeletal muscle mass index compared to baseline levels, expressed as 100% (leucine group: 97.09 ± 3.24 vs. placebo group: 102.75 ± 2.29; *p* = 0.168) or for handgrip strength (leucine group: 94.44 ± 2.88 vs. placebo group: 101.33 ± 8.22; *p* = 0.437). The significant change in walking time led to an increase in the prevalence of subjects meeting the walking time sarcopenia criterion in the control group (*p* = 0.027). Percentage changes of calf perimeters after leucine administration were 97.95 ± 1.19 for the leucine group vs. 99.59 ± 1.10 for the placebo group; *p* = 0.325. The same trend was observed in the arm perimeter, with no differences at baseline or after treatment, or in the percentage change between groups (leucine group: 101.58 ± 1.48 vs. placebo group: 99.81 ± 2.17; *p* = 0.505). There were also no differences between groups before and after treatment when we analyzed these variables as dichotomous in the presence or absence of sarcopenia, according to the cut-off points established by Setiati et al. 2010 [26]. Similarly, no differences between groups were found when analyzing changes in the fat mass index (*p* = 0.45) or MNA total score (*p* = 0.27) or the risk of malnutrition (*p* = 0.18) based on a cut-off score < 24 in the MNA screening test [24].

There was no difference between the groups (*p* = 0.929) when analyzing walking speed in terms of the use of walking aids (elderly people who can walk independently or who require the use of a cane or a walker). When the level of exercise carried out by the subjects was studied, no differences were observed between the subjects in the leucine and placebo groups. In addition, no significant correlations were found between either mild (*p* = 0.163, *rho* = 0.238, Spearman test) nor moderate (*p* = 0.481, *rho* = 0.121, Spearman test) physical activity by IPAQ and walking speed. These results suggested that the improvement in walking time was not associated with the amount of auto-referred physical activity.

### 3.4. Effect of Leucine Supplementation on Sarcopenia Muscle Respiratory Criteria

No differences were observed between the two groups at baseline or after the intervention for the respiratory function parameters established as determinants of the presence of respiratory sarcopenia. The baseline values of MIP were LG: 29.85 ± 3.05 vs. PG: 29.62 ± 3.05; *p* = 0.958; the MEP values were LG: 50.44 ± 5.14 vs. PG: 59.30 ± 3.79; *p* = 0.188 and the PEF values were LG: 2.59 ± 0.26 vs. PG: 2.66 ± 0.27; *p* = 0.858. Following the intervention, the MIP values were LG: 31.89 ± 3.17 vs. PG: 26.75 ± 3.62; *p* = 0.291, MEP values were LG: 46.91 ± 4.68 vs. PG: 45.83 ± 4.90; *p* = 0.875 and the PEF values were LG: 2.73 ± 0.26 vs. PG: 2.39 ± 0.20; *p* = 0.325. The changes in values of respiratory sarcopenia expressed as a percentage of the baseline levels are presented in Figure 2B. There was a significant difference in MEP between the leucine and placebo group, with a significant decline in expiratory muscle strength in the placebo group (LG: 99.05 ± 6.80 vs. PG: 77.67 ± 5.85; *p* = 0.026, Cohen’s d = 3.37, effect size r = 0.86). There were no statistical differences between the groups for MIP (LG: 116.32 ± 9.44 vs. PG: 93.94 ± 9.40; *p* = 0.105) or PEF values (LG: 110.75 ± 6.61 vs. PG: 100.46 ± 6.24; *p* = 0.272). Given the relevance of bronchodilator intake and smoking, it was decided that we would study the results obtained in those terms. However, no differences were found for any of the parameters studied according to the use of bronchodilators (*p* = 0.287; *p* = 0.242; *p* = 0.792) or tobacco consumption (*p* = 0.379; *p* = 0.495; *p* = 0.380), for MIP, MEP and PEF respectively.

### 3.5. Effect of Leucine Administration on Blood Analytical Parameters

Routine blood analysis was performed, e.g., hemogram and markers of hepatic function (transaminases), renal function (creatinine), lipid profile, glucose, and total proteins, ions and vitamin D (as total 25(OH)D). In addition, we measured markers of inflammation such as the C-reactive protein, TNF-α, and IL-6. As shown in Table 2, no significant differences were observed between the leucine and placebo groups (significance at *p* < 0.05). No significant differences were observed in these analytical parameters between the two groups at baseline and after completion of the trial (Table 2).

## 4. Discussion

The most effective interventions to treat sarcopenia or decrease its worsening over time are currently based on the practice of physical exercise such as resistance training and the administration of some nutritional supplements [27,28]. However, many older people are sedentary and either cannot (social barriers and family support, functional and cognitive impairments) or do not want to exercise. In these cases, nutritional interventions remain the most promising measure for delaying the progression of sarcopenia and preventing its adverse consequences, such as falls, mobility loss, and a bed-to-sofa lifestyle. The supplementation of whey protein which contains high amounts of the amino acid leucine or a mixtures of branched-chained amino acids with or without other nutritional supplements have been the most common interventions tested for treating sarcopenia in community-dwelling older individuals [13,14,15,29]. However, the effect of leucine alone, the amino acid shown to stimulate muscle synthesis and reduce its turnover in the most powerful way [30,31,32,33,34,35,36], has seldom been evaluated and no studies have been performed in institutionalized older individuals showing high rates of sarcopenia, functional impairment and of comorbidities all promoting muscle loss. In addition, most of the studies of leucine-enriched protein supplementation have been conducted in community-dwelling individuals [14] and the effects on institutionalized older individuals may present different results, as the rate of comorbidities and functional impairment is higher in institutionalized individuals. Supplementation with leucine has small but significant effects on muscle mass index, not because it improved these parameters in those individuals supplemented with leucine, but because in the placebo group there was a decline in this sarcopenia parameter. These results could mean that leucine supplementation helped to maintain lean muscle mass over longer periods of time. In addition, the beneficial effect of keeping the muscle mass stable over time may be also related to the improvement of nutritional status achieved among the individuals in the leucine-treated group because at the baseline, 38% of the individuals in this group had a risk of malnutrition (as assessed by the MNA test) and supplementation with leucine could have afforded some benefit in this subgroup of malnourished patients. In contrast, in the placebo group the risk of malnutrition occurred in 23% of the sample and this difference is unlikely to have accounted for the loss of muscle mass observed in the placebo group over time. The changes in lean mass do not parallel the changes in muscle strength, and no differences in this parameter were observed between the two groups. Our results for the lack of effect on muscle mass and strength in the leucine group are consistent with two studies performed on older men, in which leucine supplementation (7.5 g/day) did not alter muscle mass or strength in either healthy elderly men (mean age 71 years) [37] or older men with type 2 diabetes [38]. In contrast, we observed a significant improvement in physical performance (walking time) following leucine supplementation compared to the baseline values, whereas no effect was observed in the control (placebo) group. A possible explanation for the beneficial effect on physical performance in leucine-supplemented individuals might be related to the fact that physical performance is not only mediated by an efficient and proper lean mass, but also by the central and peripheral nervous system, modulating motor activity and muscular function through the neuromuscular unit [39,40]. In fact, the beneficial effects on neuromuscular activation precede the beneficial effects on increasing muscle mass in response to resistance training [41]. In most studies, there have been no clear parallels between the positive effect on increasing or maintaining lean mass and the positive effect on muscle strength or physical performance, confirming the crucial role of the nervous system in restoring muscle strength in age-related sarcopenia; therefore, the presence of a strong effect on sarcopenia requires both beneficial effects on muscles and the nervous system. Only one study reported the beneficial effect of leucine supplements on both walking speed and muscle strength in older individuals living in nursing homes, but, in that study, leucine was administered with other essential amino-acids (3 g/day), vitamin D and with medium-chain triglycerides (6 g) [30]. The co-supplementation could have benefited the muscle strength, which is not affected by leucine supplementation alone, as shown in the present RCT.

Interestingly, in older individuals with low physical activity, the effect of leucine supplementation on physical performance resembled those achieved by physical exercise in individuals with similar features, as in the present study. In fact, a recent longitudinal intervention study of elderly people living in a nursing home that evaluated the effect of a resistance training program on sarcopenia and functionality of older individuals living in nursing homes found a significant improvement in physical performance, balance, and gait speed in leucine-treated individuals [42]. Najafi et al [43], in an RCT conducted in sixty-three older adults, showed that a fun physical activity (including strength, balance, endurance, and walking activities in the form of rotational movements of the hands with plastic balls) reduced the progression of sarcopenia by improving balance, increasing distances walked and, in this case, also strengthening muscles. The risk of respiratory complications and infections increases substantially in old age, which may be due in part to sarcopenia of the diaphragm muscle and other respiratory muscles, mainly reducing their force-generating capacity and impairing the ability to perform expulsive non-ventilatory motor behaviors critical for airway clearance [44,45]. We evaluated respiratory muscle function, and we found that leucine administration improved all the respiratory muscle parameters, but the effect was significant for the MEP. Several reports have demonstrated that nutritional intervention can improve respiratory muscle function in patients with chronic obstructive pulmonary disease (COPD) [46,47,48], and even simple nutritional supplementation in malnourished individuals can improve these parameters in COPD patients as well [46,49,50]. This is the first report showing that leucine supplementation can increase MEP function, and these effects cannot be attributed to a different number of patients with COPD and the corresponding use of bronchodilator drugs between the two groups (four individuals in the leucine group and three individuals in the placebo group). The improvements in both walking time and respiratory capacity could be related to each other through the known stimulating effect of leucine on the mammalian target of rapamycin (mTOR) in skeletal muscles [51]. A recent study showed that the supplementation of leucine in diet-induced obese mice significantly improved mitochondrial function in skeletal muscles through the activation of mTOR1 [52]. The same effect was also observed in the diaphragm of sedentary and exercised rats [53]. Muscle mass did not increase in both animal studies, which is also consistent with the results of our clinical trial. Finally, the beneficial effects of leucine supplementation in this trial seem unrelated to changes in inflammatory markers in blood such as leucocyte counts, reactive C- protein, IL-6 and TNF-alpha, which have all been associated with sarcopenia [41,54,55,56]. However, the role of other molecules in the complex inflammatory cascade cannot be ruled out. We assessed the concentration of vitamin D in blood (as total 25(OH)D) because it displays important factors for assessing the physiology of muscle mass and function and sarcopenia [57]. The current guidelines [58] establish vitamin D deficiency when serum 25-hydroxyvitamin D (25(OH)D) levels are less than 20 ng/mL (50 nmol/liter). In our study, the mean levels were around 40 ng/mL and none of the individuals had levels below 20 ng/mL. A report performed in the US population (referring to the 2003–2006 period) stated that 9% had low serum 25-hydroxyvitamin D concentration (<50 nmol/L) [59,60]. The reason for the lack of vitamin D deficiency can be due to the benefits of institutionalization, such as following a controlled diet from a nutritionist/dietitian (which likely ensures an adequate vitamin D intake). In addition, in the nursing homes in Spain, supplementation of vitamin D in deficient individuals is performed by medical doctors working in these institutions. In our study, there were four individuals receiving vitamin D in the leucine group and five individuals in the placebo group. In all cases, vitamin D administration and calcium were prescribed as anti-osteoporotic treatments before and during the trial. No differences were observed in the outcomes comparing individuals under vitamin D supplementation. 

The study presents several limitations. There were eight participants (16%) that did not wish to continue for unknown reasons, which could have biased the results compared to an intention-to-treat analysis. However, baseline characteristics of the dropouts were comparable to those subjects included in the final analysis, and the dropout rate was not significantly different between the groups. Information about participants’ health was not collected on a weekly basis; therefore, it is unknown when participants first showed improvement based on the sarcopenia criteria. This study focused on changes in muscle strength and function, although muscle size was not assessed directly by computer tomography or magnetic resonance imaging. Thus, it cannot be ruled out that changes in lean muscle mass could have been detected with these more sophisticated measurements. One of the primary outcome measurements, handgrip strength, is a well-validated proxy measurement for lower body strength, but is less sensitive than other measures of strength [61]. Further research is needed to investigate sensitive and specific outcomes for sarcopenia, such as lower extremity strength and function. The population sample enrolled in the present study was characterized by low physical activity and most had a sedentary lifestyle. As shown in Table 1, assistance with walking (i.e., the aid of a walker) was required by 31.8% in the placebo group and 35% in the leucine group. In all the participating nursing homes, there was a gymnasium in which the residents could exercise. Only three participants in the placebo group and two participants in the leucine-group performed regular exercise, which involved stationary cycling for approximately 30 min per day at a low intensity. All other participants attended the gymnasium to undertake psychomotor activities at low intensity such as, e.g., chair-based exercises, ball throwing and catching. However, participants were stratified for randomization physical activity from the results of the self-administered IPAQ. It should be noted that no participants performed vigorous physical activities or moderate physical activities. Most of them walked in the gardens of the nursing homes and had a fairly sedentary lifestyle. Future studies should include the use of accelerometers for the evaluation of physical activity, as well as objective measurements of cardiorespiratory fitness, e.g., treadmill or cycling tests. The advantage of the present study design is that it can easily be implemented in the nursing homes during periods when exercise is neither possible nor feasible, or simply when residents do not wish to exercise. Even the best approaches for treating sarcopenia or delaying its progression over time are currently based on both physical exercise and nutritional supplementation [27,28,62,63,64]; our RCT demonstrated that this nutritional intervention does not negatively affect the rate of loss in skeletal muscle, but does improve physical performance and respiratory muscle function, which could also be significantly beneficial for institutionalized individuals who present with a huge burden of comorbidities and functional impairments.

## Figures and Tables

**Table 1:** Psycho-geriatric baseline characteristics of participants enrolled in the study.

	PLACEBO Mean Values (± Standard Deviation or Percentage)	LEUCINE Mean Value (± Standard Deviation or Percentage)	*p* Value
Deambulation	Independent 68.2% Walker 31.8 %	Independent 65.0% Walker 35.0%	0.14
Barthel index score	78.0 ± 21.6	78.7 ± 21.2	0.92
MMSE (Mini Mental State Examination) score	27.9 ± 5.1	29.3 ± 4.0	0.337
Comorbidities (Charlson index)	5.4 ± 2.1	5.1 ± 1.7	0.69
Nutritional status (Mini Nutritional assessment)	Normal 77.3% Malnutrition risk 22.7%	Normal 63.2% Malnutrition risk 36.8%	0.32
Body mass index (kg/m^2^)	29.1 ± 5.7	28.9 ± 7.1	0.91
Fat (% of body weight)	42.9 ± 15.7	40.4 ± 8.9	0.57
Fat (kg)	29.8 ± 11.1	28.3 ± 10.9	0.69
Fat mass index (Kg/m^2^)	11.6 ± 5.2	12.0 ± 5.5	0.79
Calf perimeter	Men: 35.3 ± 5.3 Women: 35.8 ± 5.6	Men: 34.7 ± 5.0 Women: 33.5 ± 4.2	*p* = 0.62 *p* = 0.24
Arm perimeter	Men: 28.7 ± 4.3 Women: 29.11 ± 3.2	Men: 29.2 ± 4.1 Women: 28.5 ± 3.5	*p* = 0.46 *p* = 0.62
Lean mass index Janssen (kg/m^2^)	10.4 ± 3.4	8.3 ± 1.8	0.02
Muscular handgrip strength (kg)	19.2 ± 8.6	16.3 ± 8.5	0.28
Walking time (sec)	10.4 ± 12.5	10.4 ± 10.5	1.00

**Table 2:** Blood analysis and hemogram after treatment with placebo group (PG) or leucine group (LG).

Variable	PG	LG	*p* value
Leukocytes (× 10^3^/µL)	7.5 ± 0.4	7.5 ± 0.6	0.97
Neutrophils (× 10^3^/µL)	4.4 ± 0.2	4.5 ± 0.2	0.95
Lymphocytes (× 10^3^/µL)	2.3 ± 0.1	2.2 ± 0.2	0.94
Monocytes (× 10^3^/µL)	0.53 ± 0.03	0.54 ± 0.02	0.98
Eosinophils (× 10^3^/µL)	0.22 ± 0.04	0.22 ± 0.05	1.00
Basophils (× 10^3^/µL)	0.03 ± 0.01	0.03 ± 0.01	1.00
Platelets (× 10^3^/µL)	240 ± 38	234 ± 32	0.82
Erythrocytes (× 10^6^/µL)	5.0 ± 0.7	4.9 ± 0.4	0.86
Hemoglobin (g/dL)	12.3 ± 1.0	12.9 ± 1.2	0.97
Glucose (mg/dL)	97 ± 13	93 ± 12	0.88
Urea (mg/dL)	42 ± 5	44 ± 7	0.88
GOT (U/L)	28 ±4	27 ± 3	0.86
GPT (U/L)	23 ± 2	22 ± 4	0.94
HDL cholesterol (mg/dL)	44 ± 5	43 ± 7	0.96
LDL cholesterol (mg/dL)	122 ± 10	125 ± 14	0.71
Triglycerides (mg/dL)	127 ± 24	131 ± 18	0.70
Total Proteins (g/dL)	7.1 ± 0.3	7.2 ± 0.5	0.84
Creatinine (mg/dL)	0.81 ± 0.12	0.82 ± 0.13	0.91
Calcium (mg/dL)	8.5 ± 0.7	8.5 ± 0.8	1.00
Sodium (mEq/L)	140 ± 3	141 ± 4	0.91
Potassium (mEq/L)	4.5 ± 0.9	4.5 ± 0.7	0.96
C-reactive Protein (mg/L)	5.1 ± 1.8	5.6 ± 1.4	0.41
TNF-α (pg/mL)	2.8 ± 0.3	3.2 ± 0.5	0.42
IL-6 (pg/mL)	2.0 ± 0.3	2.4 ± 0.6	0.31
Vit- D (25OHD) (ng/mL)	40.1 ± 5.1	41.2 ± 5.2	0.92

GOT: glutamic oxaloacetic transaminase; GPT: Glutamic-pyruvate-transaminase; HDL: high-density lipoprotein; LDL: low-density lipoprotein.
